# Fire needle therapy for recurrent skin and soft tissue infections: a case report and literature review

**DOI:** 10.3389/fmed.2026.1881440

**Published:** 2026-09-30

**Authors:** Wenqing Xu, Zuohang Zhou, Haixu Jin, Su Fu, Chao Wang, Chuanlong Zhou

**Affiliations:** 1 The Third Clinical College of Zhejiang Chinese Medical University, Hangzhou, China; 2 Department of Acupuncture and Moxibustion, The Third Affiliated Hospital of Zhejiang Chinese Medical University, Hangzhou, China

**Keywords:** antimicrobial resistance, bloodletting therapy, case report, cupping therapy, fire needle therapy, recurrent skin and soft tissue infections

## Abstract

**Background:**

Recurrent skin and soft tissue infections (RSSTIs) are challenging infectious diseases with a clinical incidence ranging from 7% to 45%, imposing sustained physical and psychological burdens and significant economic pressure on healthcare systems. For purulent infections, current Western medicine primarily relies on antibiotics and incision and drainage. Still, it faces limitations including increasing antibiotic resistance and surgical trauma, which further elevate the risk of RSSTIs. Fire needle therapy, as an external treatment in Traditional Chinese Medicine, may improve local microcirculation, facilitate drainage of purulent material, and promote wound healing. Due to its operational simplicity and lack of drug dependency, it offers a novel approach for treating RSSTIs.

**Case description:**

A 70-year-old male patient presented with recurrent redness, swelling, and pus discharge on the back for 6 months, with exacerbation over the preceding 2 days. The patient was diagnosed with RSSTIs and had previously undergone surgical incision and drainage, but with poor efficacy and recurrent episodes. After 3 sessions of fire needle therapy, the wound healed completely, with no recurrence during the 1-year follow-up period and no adverse events throughout the treatment.

**Conclusion:**

This case suggests that fire needle therapy may represent a potential adjunctive approach for the management of RSSTIs in selected patients. However, conclusions regarding efficacy and safety cannot be drawn from a single case, and further controlled clinical studies are required.

## 1 Introduction

Skin and soft tissue infections (SSTIs) comprise a heterogeneous group of infectious conditions involving the skin and underlying subcutaneous tissue, fascia, or muscle. They include conditions such as furuncles, carbuncles, cutaneous abscesses, and cellulitis and range from superficial infections to severe necrotizing infections. Common clinical manifestations include erythema, swelling, warmth, tenderness, and pain. Severe cases may be accompanied by systemic manifestations (1). Notably, a significant proportion of patients with SSTIs experience recurrence, with at least 16.7% experiencing one or more recurrent episodes within 1 year (2). Recurrent skin and soft tissue infections (RSSTIs) not only exacerbate patients’ physical suffering and anxiety but also significantly increase antibiotic usage frequency and healthcare expenditure, making them a clinical challenge. We report a patient with RSSTIs and multiple comorbidities who achieved clinical resolution following fire needle therapy, suggesting that fire needle therapy may have potential as an adjunctive treatment option for RSSTIs. However, current evidence remains limited and requires further clinical validation.

## 2 Case presentation

### 2.1 Clinical presentation

A 70-year-old male patient presented to our hospital on August 26, 2025, complaining of recurrent redness, swelling, and pus discharge on his back for 6 months, with exacerbation over the preceding 2 days. Laboratory examination upon admission showed: white blood cell count 11.4 × 10⁹/L, with a neutrophil percentage of 82%. Computed tomography (CT) revealed inflammatory infiltration in the subcutaneous tissue of the back (Figure 1), while ultrasonography showed a hypoechoic area measuring approximately 4.3 cm × 1.0 cm. Physical examination: an approximately 8 × 7 cm area of redness and infiltration was visible beside the spinal column on the back, with relatively clear boundaries, surrounding skin erythema, increased skin temperature, obvious tenderness, and a central mass with two visible pus points at the center, without obvious fluctuation. After evaluating all findings, we diagnosed him with RSSTIs. The patient was an elderly male with a history of ankylosing spondylitis, cervical fracture surgery, and lung surgery. He was long-term bedridden and presented with concurrent hypoproteinemia and sarcopenia. He had previously received surgical debridement, silver-ion dressing changes, and nutritional support at another hospital (Figure 2), but symptoms recurred, and the skin infection persisted, prompting him to seek further treatment at our hospital.

**Figure 1:**
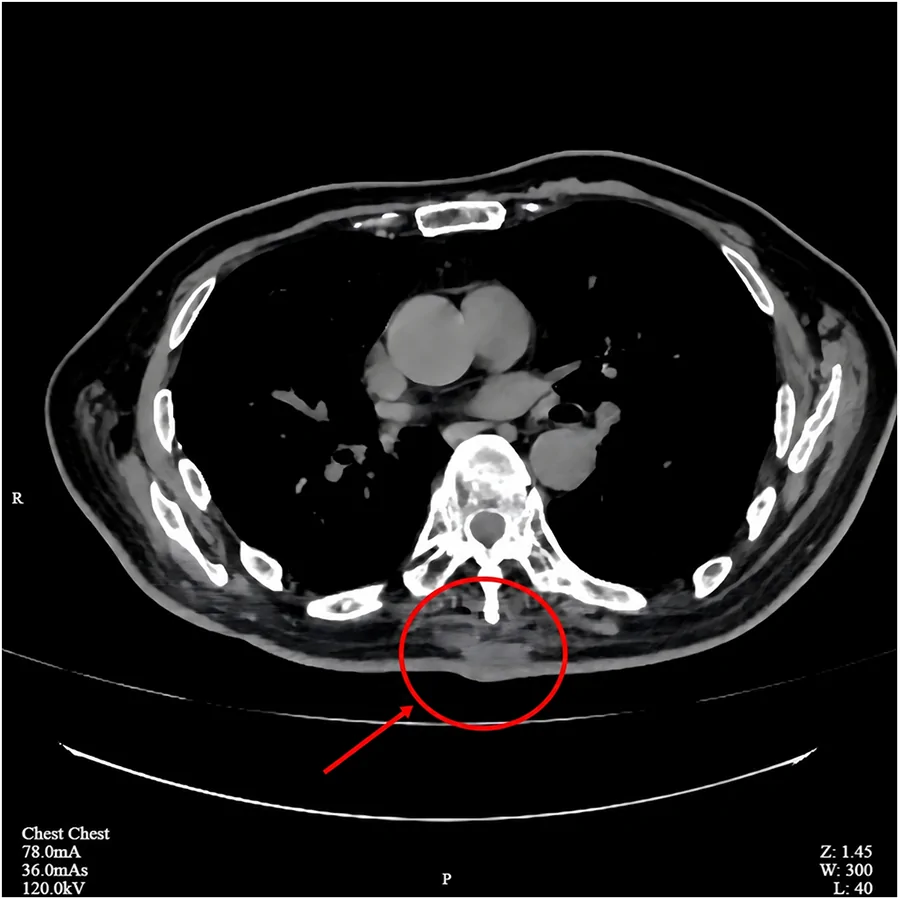
Baseline CT image showing a low-attenuation area in the subcutaneous tissue of the back (arrow).

**Figure 2:**
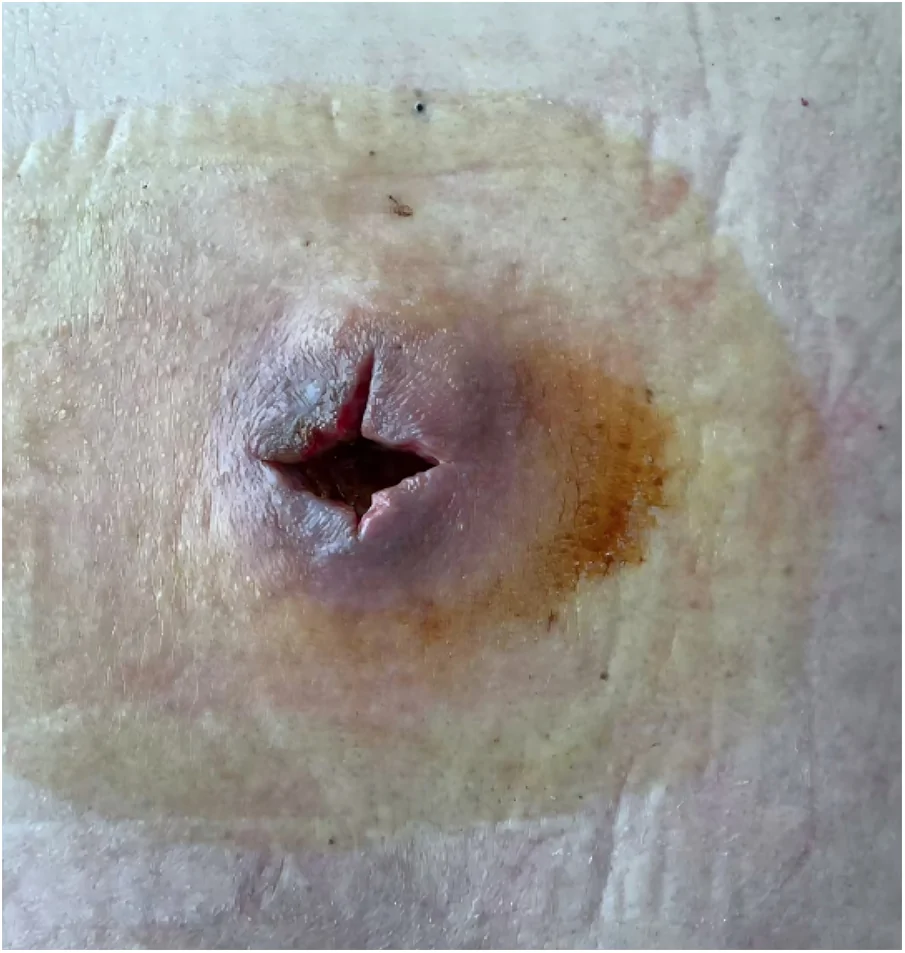
Wound condition after the first incision and drainage surgery.

### 2.2 Acupuncture treatment

On the initial visit of August 26, 2025, we developed a treatment plan centered on fire needle therapy. The main operation sites were selected as the redness-swelling margin on the back and the Ah-shi points (center of the mass). A 0.4 mm × 13 mm fine fire needle and a 0.6 mm × 50 mm thick fire needle were used. The patient was placed in a prone position with the affected area fully exposed. After routine disinfection, the practitioner held the fine fire needle in the right hand, heating the anterior 1/3 of the needle body to white-hot in the outer flame of an alcohol lamp, then quickly and shallowly piercing the redness margin to a depth of approximately 0.5 cm. Subsequently, the thick fire needle was rapidly inserted 1 cm into the Ah-shi point, with slight twisting to expand the pus drainage channel, and yellowish-white viscous pus was observed flowing out from the needle hole. Cupping therapy was immediately applied to the needle-pierced area, maintaining negative pressure suction for 5–10 min until fresh blood was visible. After the procedure, the wound was cleaned again, compressed with sterile gauze, and the patient was instructed to keep the area dry. Thereafter, if exudation was significant, dressings were changed once daily. All fire needle and cupping operations were performed by a licensed acupuncturist with many years of clinical experience.

### 2.3 Clinical outcomes

After the first treatment, the area of local redness and swelling decreased, exudation decreased, and the patient reported marked pain relief. On August 30, 2025, a second fire needle treatment was performed; the amount of discharged fluid was substantially reduced, the wound contracted, and tenderness decreased. By the third visit on September 3, 2025, the wound had continued to contract, and local skin temperature basically returned to normal. On September 9, 2025, redness, swelling, heat, and pain had disappeared, and the wound was almost completely healed. Thereafter, routine dressing changes were maintained twice weekly. At the follow-up on September 25, 2025, the wound had completely healed, leaving only a superficial scar, with no clinical signs of infection. At the 1-year follow-up on September 9, 2026, conducted through patient report and photographic documentation, the wound remained clinically healed. No recurrent redness, swelling, pain, or purulent discharge was reported, and the patient had received no further antimicrobial therapy or surgical intervention. No adverse events occurred throughout the entire treatment process. The specific treatment course and changes in wound healing are shown below (Figure 3).

**Figure 3:**
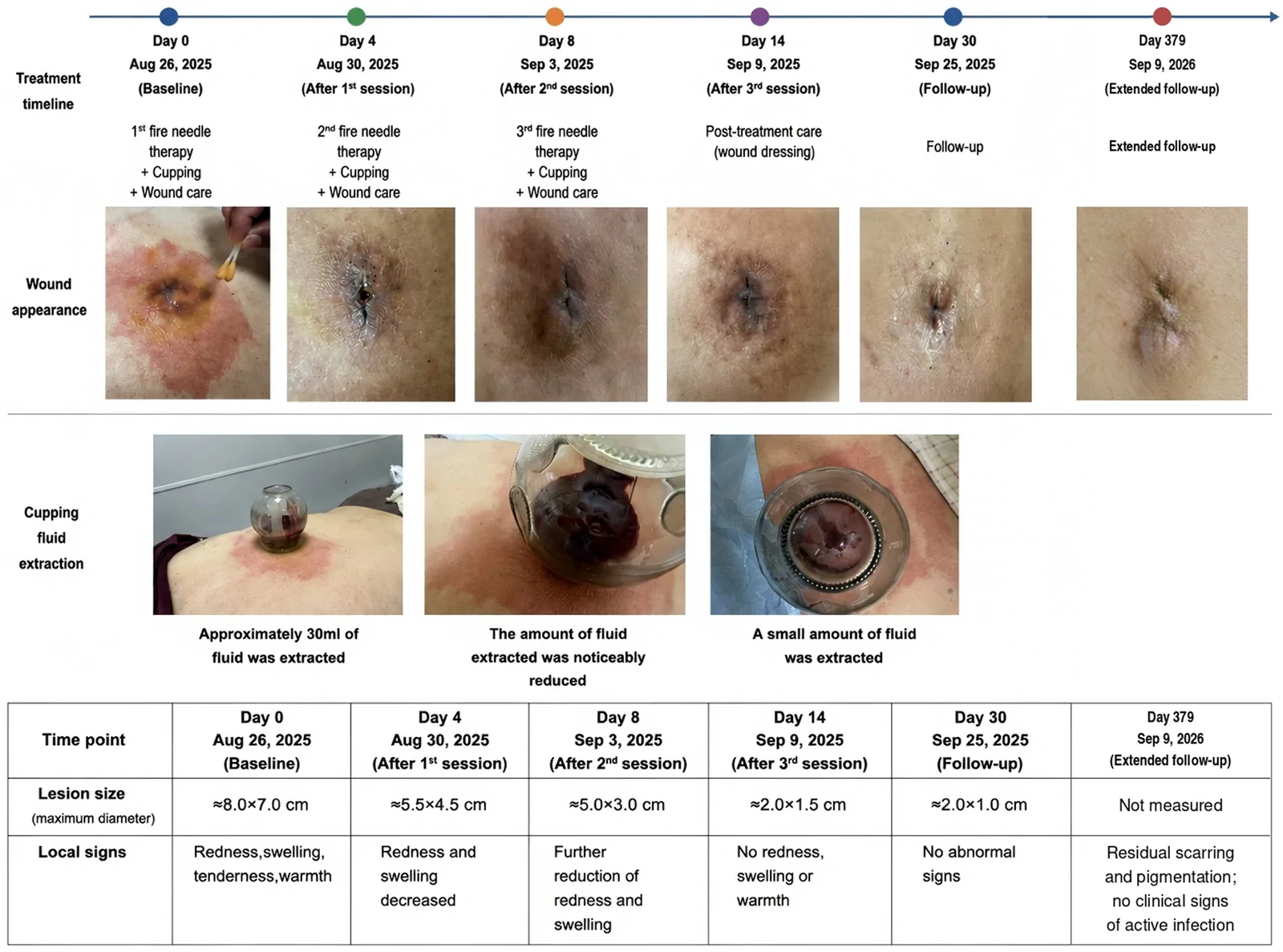
Integrated clinical timeline showing wound healing progression, lesion size changes, and treatment course following fire needle therapy.

## 3 Discussion

### 3.1 Clinical characteristics and treatment challenges of RSSTIs

SSTIs are the most common local infections (3), accounting for up to 14 million outpatient visits and nearly 900,000 hospitalizations annually in the United States, with infection-related costs rising year by year (4). Studies show that the rate of RSSTIs ranges from 7% to 45%, and obesity, diabetes, malignancy, neutropenia, and immunosuppressive status are the most critical systemic promoting factors (5). The pathogen spectrum of SSTIs is dominated by Gram-positive cocci, with Staphylococcus aureus and Streptococcus species being the primary causative bacteria (6). Due to its strong colonization ability and intrinsic resistance (7), Staphylococcus aureus occupies a dominant position among RSSTIs pathogens. Approximately 20%–30% of the population are persistent asymptomatic carriers of Staphylococcus aureus in the nasal vestibule, with another approximately 30% being intermittent carriers, nasal colonization has been confirmed as an independent risk factor for infections at other body sites (8). In recurrent patients, the same strain can be detected colonizing the pharynx, perineum, hands, and skin surface (9), and this multi-site “colonization network” significantly increases the difficulty of completely eradicating Staphylococcus aureus. Staphylococcus aureus infections exhibit “clustering” within families (10), which is one of the environmental factors contributing to RSSTIs. The elderly population, due to skin cell aging, reduced collagen fiber synthesis, and accelerated degradation, combined with multiple chronic comorbidities and malnutrition, are prone to stubborn and recurrent infections once formed, facing high RSSTIs risk (11). For purulent infections, incision and drainage combined with antibiotic therapy is the current standard treatment (12), but surgical procedures carry greater trauma and higher complication risks. Topical antibiotics are recommended as first-line treatment for various SSTIs indications (13), but in clinical practice, physicians tend to prescribe oral antibiotics; this use of systemic antibiotics in the absence of systemic infection symptoms accelerates the trend of microbial resistance (14).

### 3.2 Case interpretation and diagnostic basis in the present case

The diagnosis of RSSTIs in this case was established based on a combination of clinical manifestations, laboratory findings, and imaging features. The patient presented with recurrent localized redness, swelling, tenderness, and purulent discharge over a 6-month period, accompanied by leukocytosis with neutrophil predominance. Imaging examinations, including CT and ultrasonography, revealed subcutaneous inflammatory infiltration and a hypoechoic lesion consistent with a localized suppurative process. Collectively, these findings supported a clinical diagnosis of RSSTIs.

However, the absence of microbiological culture and histopathological confirmation limits etiological certainty. In elderly patients with multiple comorbidities and prolonged immobilization, recurrent soft tissue lesions may represent a spectrum of overlapping inflammatory and infectious conditions. Therefore, differential diagnosis should be carefully considered.

First, an infected epidermoid cyst should be excluded, as it commonly presents as a recurrent subcutaneous nodule with episodic inflammation and purulent discharge (15). However, in the present case, no well-defined cystic capsule or typical keratinous content was identified on imaging or during clinical assessment, making this diagnosis less likely.

Second, a chronic or encapsulated abscess should be considered. Chronic abscesses may persist with intermittent drainage and incomplete resolution following incision and drainage (16). The patient's history of prior surgical debridement with recurrence is compatible with this possibility. However, the diffuse inflammatory infiltration pattern on imaging and the absence of a well-formed encapsulated cavity argue against a fully organized chronic abscess (17).

Third, hidradenitis suppurativa (HS) is an important differential diagnosis in recurrent suppurative lesions. HS typically involves apocrine gland-bearing areas such as the axilla and groin and is characterized by sinus tract formation and multiple interconnected lesions (18). The lesion location on the back and absence of typical double comedones or sinus tract networks make HS unlikely in this case.

Fourth, pressure-related soft tissue infection, including pressure ulcer–associated infection, was also considered given the patient's reduced mobility. However, the lesion was not located over a typical bony prominence (19), and there were no clinical or imaging features suggestive of pressure injury such as overlying skin breakdown or necrotic ulceration, reducing the likelihood of this etiology.

Fifth, postoperative scar-related infection or sinus tract formation should also be considered due to the patient's history of prior surgical interventions (20). However, there was no clear evidence of persistent fistula or deep tract continuity on imaging, making this diagnosis less probable.

Overall, RSSTIs represented the most consistent clinical diagnosis but remained unconfirmed microbiologically. This highlights the importance of integrating microbiological, imaging, and histopathological data in future studies to improve diagnostic precision and reduce potential misclassification.

### 3.3 Evidence base and clinical implications of fire needle therapy in infectious skin diseases

Previous studies have demonstrated that fire needle therapy has unique advantages in treating various infectious and inflammatory skin diseases. In terms of minimal invasiveness and cosmetic needs, its trauma is significantly less than that of traditional incision and drainage, making it especially suitable for patients with high cosmetic demands. Zhang et al (21) reported that fire needle therapy for simple skin abscesses achieved complete remission or significant remission with no recurrence during follow-up, resulting in more ideal cosmetic outcomes than surgical debridement. Similarly, pediatric case series have suggested that fire needle-based interventions may promote wound healing within 4–14 days without scarring (22). In reducing recurrence rates, Zhang et al (23) reported that compared with chemical peels alone, fire needle adjuvant chemical peel therapy improved the total effective rate and reduced recurrence rates. Tang et al (24) found that fire needle combined with aminolevulinic acid-based photodynamic therapy (ALA-PDT) for moderate-to-severe acne was superior to ALA-PDT alone, not only significantly improving the clinical effectiveness rate but also effectively reducing the Global Acne Grading System (GAGS) score and recurrence rate of acne. In terms of immune and anti-inflammatory regulation, fire needle combined with cupping therapy was reported to reduce the incidence of postherpetic neuralgia and was associated with changes in the balance between T helper 17 (Th17) cells and regulatory T (Treg) cells (25). Collectively, these studies suggest the clinical potential of fire needle therapy in infectious skin diseases. However, the overall quality of evidence remains limited, with most data derived from case series, small clinical studies, or adjunctive treatment designs. Therefore, its role in infectious skin disease management requires further validation through well-designed randomized controlled trials.

### 3.4 Potential mechanisms underlying the clinical improvement following fire needle therapy

The present case demonstrated satisfactory wound healing following fire needle therapy. However, as a single case report without microbiological or biological assessments, the mechanisms responsible for the observed clinical improvement cannot be determined. Based on previous experimental and clinical evidence, several potential mechanisms may be considered.

First, minimally invasive fire needle puncture combined with cupping-assisted drainage may facilitate the removal of local exudate. Compared with conventional surgical incisions, fire needling creates relatively smaller wounds, which not only aids in local wound management but also effectively avoids unnecessary damage to the surrounding healthy tissue. This process may convert a chronic wound environment into an acute inflammatory state (26), triggering platelet adhesion, activation, and aggregation and the release of growth factors such as vascular endothelial growth factor (VEGF), fibroblast growth factor 2 (FGF-2), and platelet-derived growth factor (PDGF), which promote angiogenesis and tissue repair (27). Additionally, thermal stimulation can directly denature and coagulate microbial proteins (28), giving fire needle therapy a bactericidal effect that may be superior to mechanical debridement alone.

Second, fire needle therapy may facilitate wound healing by modulating the local inflammatory microenvironment. Thermal stimulation of fire needle therapy has been reported to improve local blood and lymphatic circulation, reduce blood viscosity, decrease the expression of oxidized lipoproteins, and alleviate tissue ischemia and hypoxia (29), thereby creating a more favorable environment for tissue repair. Macrophage polarization plays an essential role in the transition from inflammation to tissue repair. Inflammation stimulates macrophage polarization into pro-inflammatory (M1) or anti-inflammatory (M2) phenotypes (30). Experimental studies have suggested that fire needle therapy can influence macrophage polarization and inflammatory balance, potentially facilitating wound healing (31). The hyperemic response after cupping can synergize with fire needle therapy to promote macrophage polarization toward the anti-inflammatory M2 phenotype, thereby providing numerous local and systemic health benefits (32). Also, fire needle therapy can activate the body's natural regulatory mechanisms, promote granulation tissue formation, and accelerate healing (33).

In terms of drug safety, long-term or repeated use of systemic antibiotics in elderly and immunocompromised populations carries many hidden dangers, including disruption of gut microbial homeostasis, increased risk of hepatorenal toxicity, and accelerated selection of drug-resistant strains (34, 35). In this case, no additional systemic antibiotics were used during this treatment episode, not only avoiding the above drug-related risks but also reducing the patients’ psychological burden of long-term drug dependency, offering unique advantages.

However, it should be acknowledged that the proposed mechanisms were not directly assessed in this patient and therefore remain hypothetical. The clinical course indicates a possible adjunctive contribution of fire needle therapy in RSSTIs, but robust evidence from controlled clinical studies is required to establish its therapeutic value and safety.

### 3.5 Limitations and future research directions

This case has several important limitations. First, microbiological confirmation including wound culture and pathogen identification was not performed, preventing identification of the causative organism and assessment of antimicrobial effects. Second, no standardized objective outcome measures, such as pain scoring and inflammatory biomarkers, were systematically collected. Third, although the follow-up period was extended to 1 year, the long-term outcome was assessed primarily through patient report and photographic documentation without standardized clinical evaluation, which limited the objective assessment of recurrence. Fourth, fire needle therapy was administered in combination with cupping therapy and routine wound care, making it impossible to determine the independent contribution of fire needle therapy. Finally, as a single case report, the findings are inherently limited in generalizability and should be interpreted as hypothesis-generating. Future studies should incorporate microbiological assessment, quantitative clinical endpoints, longer follow-up, and controlled comparative designs to better evaluate the efficacy and safety of fire needle therapy in RSSTIs.

## 4 Conclusion

We report the management of an elderly patient with RSSTIs following insufficient response to conventional incision, drainage, and routine wound care. Complete clinical resolution of the lesion was observed, and no clinically evident recurrence was reported during the 1-year follow-up period. This case suggests that fire needle therapy may have potential as an adjunctive approach in selected patients with RSSTIs.

## Data Availability

The original contributions presented in the study are included in the article/supplementary material, further inquiries can be directed to the corresponding authors.
